# Taking stock of current societal, political and academic stakeholders in the Canadian healthcare knowledge translation agenda

**DOI:** 10.1186/1748-5908-2-32

**Published:** 2007-10-04

**Authors:** Mandi S Newton, Shannon Scott-Findlay

**Affiliations:** 1 Department of Pediatrics, Faculty of Medicine and Dentistry, University of Alberta, Edmonton, Alberta, Canada

## Abstract

**Background:**

In the past 15 years, knowledge translation in healthcare has emerged as a multifaceted and complex agenda. Theoretical and polemical discussions, the development of a science to study and measure the effects of translating research evidence into healthcare, and the role of key stakeholders including academe, healthcare decision-makers, the public, and government funding bodies have brought scholarly, organizational, social, and political dimensions to the agenda.

**Objective:**

This paper discusses the current knowledge translation agenda in Canadian healthcare and how elements in this agenda shape the discovery and translation of health knowledge.

**Discussion:**

The current knowledge translation agenda in Canadian healthcare involves the influence of values, priorities, and people; stakes which greatly shape the discovery of research knowledge and how it is or is not instituted in healthcare delivery. As this agenda continues to take shape and direction, ensuring that it is accountable for its influences is essential and should be at the forefront of concern to the Canadian public and healthcare community. This transparency will allow for scrutiny, debate, and improvements in health knowledge discovery and health services delivery.

## Background

The knowledge translation agenda remains at the forefront of international debate and concern, with extensive focus on the large gap that remains between research knowledge and healthcare practice. Indeed, the translation of basic scientific knowledge into clinical studies and the translation of clinical studies into improvements in healthcare practices remain two major obstacles in the knowledge translation agenda [1,2]. Much-cited studies from the US and the Netherlands suggest that 30 to 40% of patients do not receive treatment complying with current research evidence, 20 to 25% of the care provided to patients is not needed or may be potentially harmful [3-5], and that treatment implementation has occurred before being proven beneficial [6].

In healthcare and in health research, the knowledge translation agenda has gained increasing importance as a means to promote evidence-based practice and policy, with the intended goal being improved healthcare outcomes. Within the knowledge translation field, considerable theoretical and polemical discussions have transpired concerning what evidence is [7-12] and what constitutes evidence-based practice and policy [13-16]. Methods for studying knowledge translation have been developed, and studies evaluating the translation of research evidence into healthcare practices have been conducted [17-27]. There has been concomitant debate on who should be accountable for health research translation. To date, literature has focused on roles and responsibilities of key stakeholder relationships [28-37], specific roles that facilitate knowledge translation (*e.g.*, knowledge brokers, opinion leaders) [38-40], and organizational factors specific to stakeholder contexts have also been acknowledged as integral to the knowledge translation agenda [35,36,41]. Most recently, government has become a key stakeholder in the agenda with health research funding agencies from across the world (*e.g.*, Institute of Medicine, Medical Research Council, National Institutes of Health, Canadian Health Services Research Foundation, and Canadian Institutes of Health Research) developing key funding directives and statements on the importance of knowledge translation to healthcare to promote effective, timely, and responsible translation of health research results. In Canada, these directives carry key public and private investments for other stakeholders that, in turn, shape the country's knowledge translation agenda.

In this debate paper, we discuss the current knowledge translation agenda in Canadian healthcare that involves the influence of values, priorities, and people, and illustrate how each of these stakes shapes the discovery and translation of health research knowledge. We conclude with recommendations for the direction of this agenda in light of current stakeholder interests.

## Discussion

### The knowledge translation agenda in Canadian healthcare

The current healthcare research agenda in Canada is a more balanced one. There is a strong foundation in discovery of new health knowledge and its translation into the healthcare system. The research agenda prior to this was focused almost exclusively on the creation of new knowledge, with little funding emphasis on the actual implementation in practice or policy. Having this new agenda in healthcare, however, is complex; to be effective it needs to span macro (policy, funding), meso (organizational) and individual (researcher, decision-maker, consumer) levels of the health system which is itself a complex system with competing demands from multiple stakeholders. Adding to this complexity, is an agenda also greatly shaped by a degree of societal accountability (*e.g.*, return on investment of tax dollars earmarked for health research) and priorities (*e.g.*, identified needs for healthcare system improvements).

The Canadian movement for addressing how research influences the healthcare system and patient outcomes emerged in the early 1990s with calls in the literature for the adoption of an evidence-based, decision making culture throughout the healthcare system [*e.g.*, [9]]; the National Forum on Health swiftly spurred a similar response at a national level [42]. Borne out of these early developments, at the macro level, are knowledge translation agendas currently endorsed by Canada's two major health research funding agencies, the Canadian Institutes of Health Research (CIHR) and Canadian Health Services Research Foundation (CHSRF). Each agency offers their respective definition of knowledge translation. For CIHR, knowledge translation involves "... the exchange, synthesis and ethically-sound application of knowledge – within a complex system of interactions among researchers and users – to accelerate the capture of the benefits of research for Canadians" [43]. CHSRF uses the phrase knowledge transfer and exchange, defined as "... collaborative problem-solving between researchers and decision makers that happens through linkage and exchange. [It] results in mutual learning through the process of planning, producing, disseminating, and applying existing or new research in decision-making" [44].

Using these definitions, both agencies have established key funding directives to encourage the translation of health research knowledge to ultimately better influence policy and healthcare practice decisions. CIHR stresses accountability in the return on investment of tax dollars that fund Canadian health research [43,45]. The intent is clear: publicly funded health research should be carried out in the most effective way to facilitate timely translation of research findings into health and fiscal benefits. Since its establishment in 1999, CIHR has increased its funding three-fold in clinical research and twenty-fold in health systems research supporting its knowledge translation mandate [46]. Despite these funding increases, there is sentiment that additional funds need to be dedicated to continue to build capacity in the knowledge translation field, and the agency has proposed further developments to its knowledge translation portfolio [2,47]. Consistent with its definition of knowledge transfer and exchange, CHSRF focuses funding on applied health research projects and clearly emphasizes the need for established relationships between researchers, decision- and/or policy-makers to translate research findings to healthcare settings [44]. While the role of Canada's funding agencies in the knowledge translation agenda provides a transparent process of tracking health research funds and the impact/outputs of funded research, these positions greatly influence the country's research agenda and shape issues related to timing, translation ethics, and accountability.

### Judicious knowledge translation

While the translation of basic scientific 'bench' discoveries into clinical studies and the translation of clinical studies into improvements in health care practices remain two major obstacles in the health care system, there are no definitive timeframes from Canada's funding agencies to promote research advancement that addresses these limitations. Indeed, it may take years or decades before a body of research accumulates to provide an ethical and sound direction for health system impact. Further, research advances often involve the coordination of contributions from more than one scientific field (*e.g.*, basic and clinical researchers from nanotechnology, engineering, medicine, etc.). CIHR accounts for this important timing issue in its caveat of 'ethically-sound application' in its definition, but the message may not be clear enough to researchers when considered alongside the agency's expectations for knowledge translation. In a recent paper, the notion of judicious translation was brought forth by CIHR which fits well with this dilemma [2]. In their article, Graham and Tetroe stress that "while researchers are encouraged to translate the results of their studies, they need to be thoughtful about their message and who the appropriate audience is for this message" [[2]; pg 21].

We agree with this position; there is indeed an important ethical component to the knowledge translation agenda that should not be diminished in the effort to close the gap between 'bench and bedside'. The knowledge translation movement in healthcare can give rise to good-intentioned researchers, decision-makers, and policy-makers prematurely implementing evidence and/or interventions when there is an insufficient knowledge base to be confident in its impact; a concern and reality already echoed in the literature [3-6,48]. The sense of urgency to translate for public greater good and system improvements should be tempered with clear messages that translation is an ethically-bound process that should be judiciously appraised. In this sense, a distinction is made between what knowledge translation is to healthcare (*e.g.*, translating evidence into healthcare practice to promote system improvements) versus what knowledge translation is to health research (*e.g.*, translating research evidence into the scientific community via publication for scrutiny and/or translating evidence into healthcare practice for study). A natural debate that emerges from this distinction, but is debatable beyond the scope of this paper, is the application of best available evidence versus best evidence.

The emphasis by Canada's funding agencies on engaged activities between researchers and decision- and policy-makers to promote research translation into health benefits carries accountability issues and concerns regarding scope of practice. There is potential for considerable impact on these stakeholders. Little research has empirically examined the activities of Canadian health researchers, and whether these actually align with the country's current funding agendas [49]. Of concern is the potential tension between funding agency directives and the system that health researchers function in, an environment that expects researchers to ascend through the academic ranks via established publication and grant dollar benchmarks. Effort afforded to establishing connectivity with and products essential to decision- and policy-makers for translation is under-rewarded, if unrewarded, by university tenure and promotion systems carrying the potential of unintended adverse career effects [41,50]. The same situation can be afforded to decision- and policy-makers who are evaluated by performance standards that are not well-aligned with funding agency directives that encourage/expect involvement in the research process and translation efforts whose products often extend beyond formal, evaluative time spans in healthcare organizations.

To meet the contemporary demands of Canadian funding agencies and those of university tenure and promotion, researchers need to consider a portfolio that includes traditional knowledge translation expectations (scholarly outputs such as peer-reviewed publications) and applied knowledge translation activity (engaged interactions with decision- and policy-makers) [51-53]. Academic institutions' values need to evolve to become more utilitarian; knowledge discovery cannot be solely regarded and rewarded via traditional knowledge translation activity, but should extend to a more utilitarian standpoint where knowledge discovery is 'hand-in-hand' with potential implementation. The same philosophy can be applied to decision- and policy-makers who find themselves at odds with how to manage their portfolios. This potential solution, however, only targets individual accountability. Accountability targeted at the organizational level should also be expected. Within the knowledge translation agenda are calls for the recognition and examination of organizational factors (*e.g.*, leadership structure, hospital classification) and environmental factors (*e.g.*, the healthcare delivery team, organizational culture, administrative personnel) that shape the innovation implementation [24,38,39,56-58]. This call should also include the examination of institutions that employ the researchers (*i.e.*, academia) and decision- and policy-makers (*i.e.*, hospitals and government) as these stakeholders are also directly embedded in the organizational and environmental systems within the healthcare system. Employer promoted professional development and evaluation systems need to be re-examined and reconstructed to reflect current trends in the healthcare research agenda [52,53]. Professional development should include organization-created opportunities for relationship development and skill-building related to research application. In Canada, several examples exist to strengthen capacity in developing relationships between researchers and decision-/policy-makers (*e.g.*, community-university partnerships [CUP] programs) and developing leadership and skills to better use research information in the healthcare system, including SEARCH Canada (Swift, Efficient Application of Research in Community Health) [54] and the EXTRA (Executive Training for Research Application) programs [55]. These opportunities, however, need to be more consistent in the Canadian system as a means of formal markers for professional development and work scope.

## Summary

Knowledge translation in Canadian healthcare has public and private interests that are inherently served for stakeholders who have an influential role in knowledge creation, dissemination, and implementation to advance health knowledge and health services delivery. Tailoring recommendations for the knowledge translation agenda to these interests is a first step in creating accountability and transparency to allow for scrutiny, debate, and improvements in health knowledge discovery and health services delivery.

We recommend that the following need to be formally included as part of the knowledge translation debate and agenda in Canada:

1. The message that return on investment of tax dollars for healthcare research via translation to system improvements should be consistently tempered with the clear message that translation is an ethically-bound process that should be afforded to robust evidence to support its impact [2,53]. Changes to healthcare practice and policy demand consequential complex behaviour changes at many different levels necessitating strong evidence bases for the change. One only needs to look at the case of breast screening examination research to highlight the complexity and intricacy of interpreting research in a manner that guides clinical decisions, particularly when research calls accepted clinical practices into question. This process of interpretation of research is neither straightforward nor easy, but rather, involves time and developed skill to access, understand, critique, and reflect on research results in light of one's practice and experience.

2. Accountability for the knowledge translation agenda should span macro, meso, and micro levels. At the macro and meso levels, funding agencies, government (federal, provincial, municipal), healthcare organizations, and academic institutions need to align organizational directives related to knowledge translation of robust evidence. We need to begin publicly discussing what resources should be expected from employers to promote engaged knowledge translation activity, and how should these activities be recognized in work scope and career advancement. As the demand for research knowledge has become more utilitarian, in response, stakeholders will be more effectual if they adopt a process to address utilitarian complexities [41]. At the micro level, consideration should also start to be given as to how other public and private entities (*e.g.*, advocacy groups, media) can assume responsibility in the knowledge translation agenda. At this level, the peer-review process in evaluating research findings also warrants examination. The role of editors in publishing robust null/negative and replicated health research findings for peer and public scrutiny is a necessary component to the translation agenda. Publication bias involving positive results and the emphasis on publishing novel findings versus replicated studies can skew the landscape of health-related issues [53].

3. Organizational research should include an examination of institutions that employ the researchers and decision- and policy-makers as these stakeholders are also directly embedded in the organizational and environmental systems within the healthcare system. Further, organizationally-oriented research needs to include more sophisticated analytic work, such as the development of statistical models that demonstrate how the identified organizational features (*e.g.*, organizational size, organizational complexity, organizational slack, resources) interact and work.

## Conclusion

In Canada, there has been increasing pressure to demonstrate both accountability and transparency in healthcare decision-making; the translation of research to the healthcare system has been a frequently accepted strategy to accomplish these demands. However, this knowledge translation agenda has public and private interests that are inherently served for current stakeholders who have an influential role in knowledge creation, dissemination, and implementation to advance health knowledge and health services delivery. As this agenda continues to take shape and direction, ensuring that it is accountable for its influences is essential and should be at the forefront of concern to the Canadian public and health research community. This transparency will allow for scrutiny, debate and improvements in health knowledge discovery and health services delivery.

## Competing interests

The author(s) declare that they have no competing interests.

## Authors' contributions

Both MSN and SSF led manuscript formulation and writing. Both authors read and approved the final manuscript.

## References

[B1] Sung N (2003). Central challenges facing the national clinical research enterprise. JAMA.

[B2] Graham ID, Tetroe J (2007). How to translate health research knowledge into effective healthcare action. Healthc Q.

[B3] Grol R (2001). Successes and failures in the implementation of evidence-based guidelines for clinical practice. Med Care.

[B4] Schuster M, McGlynn E, Brook RH (1998). How good is the quality of health care in the United States?. Milbank Quarterly.

[B5] McGlynn EA, Asch SM, Adams J, Keesey J, Hicks J, DeCristofaro D, Kerr EA (2003). The quality of health care delivered to adults in the United States. NEJM.

[B6] Arnold SR, Straus SE (2005). Interventions to improve antibiotic prescribing practices in ambulatory care. Cochrane Database Syst Rev.

[B7] Ashcroft RE (2004). Current epistemological problems in evidence based medicine. Journal of Medical Ethics.

[B8] Bluhm R (2005). From hierarchy to network: A richer view of evidence for evidence-based medicine. Perspect Biol Med.

[B9] Evidence-Based Medicine Working Group (EBMWG) (1992). Evidence-based medicine: A new approach to teaching the practice of medicine. JAMA.

[B10] Haynes RB (2002). What kind of evidence is it that evidence-based medicine advocates want health care providers and consumers to pay attention to?. BMC Health Services Research.

[B11] Scott-Findlay S, Pollock C (2004). Evidence, research, knowledge: a call for conceptual clarity. Worldviews on Evidence-Based Nursing.

[B12] Upshur RE (2005). Looking for rules in a world of exceptions: reflections on evidence-based practice. Perspect Biol Med.

[B13] Guyatt GH, Meade MO, Jaeschke R, Cook D, Haynes RB (2000). Practitioners of evidence based care. BMJ.

[B14] Haynes RB, Devereaux PJ, Guyatt GH (2002). Clinical expertise in the era of evidence-based medicine and patient choice. ACP J Club.

[B15] Lavis JN, Ross SE, Hurley JE, Hohenadel JM, Stoddart GL, Woodward CA, Abelson J (2002). Examining the role of health services research in public policymaking. Milbank Quarterly.

[B16] Upshur RE, Tracy CS (2004). Legitimacy, authority and hierarchy: Critical challenges for evidence based medicine. Brief Treatment in Crisis Intervention.

[B17] Eccles M, Grimshaw J, Campbell M, Ramsay C (2003). Research designs for studies evaluating the effectiveness of change and improvement strategies. Quality & Safety in Health Care.

[B18] Freemantle N, Mason J, Eccles M (1999). Deriving treatment recommendations from evidence within randomized trials. The role and limitation of meta-analysis. International Journal of Technology Assessment in Health Care.

[B19] Green LW, Glasgow RE (2006). Evaluating the relevance, generalization, and applicability of research: Issues in external validation and translation methodology. Evaluation & the Health Professions.

[B20] Grimshaw J, Campbell M, Eccles M, Steen N (2000). Experimental and quasi-experimental designs for evaluating guideline implementation strategies. Family Practice.

[B21] Grol R, Grimshaw J (2003). From best evidence to best practice: effective implementation of change in patients' care. Lancet.

[B22] Titler MG (2004). Methods in translation science. Worldviews on Evidence-Based Nursing.

[B23] Bero LA, Grilli R, Grimshaw JM, Harvey E, Oxman AD, Thomson MA (1998). Closing the gap between research and practice: an overview of systematic reviews of interventions to promote the implementation of research findings. The Cochrane Effective Practice and Organization of Care Review Group. BMJ.

[B24] Dijkstra R, Wensing M, Thomas R, Akkermans R, Braspenning J, Grimshaw J, Grol R (2006). The relationship between organizational characteristics and the effects of clinical guidelines on medical performance in hospitals, a meta-analysis. BMC Health Services Research.

[B25] Farquhar CM, Stryer D, Slutsky J (2002). Translating research into practice: The future ahead. International Journal for Quality in Health Care.

[B26] Nilsen ES, Myrhaug HT, Johansen M, Oliver S, Oxman AD (2006). Methods of consumer involvement in developing healthcare policy and research, clinical practice guidelines and patient information material. Cochrane Database Syst Rev.

[B27] Oxman AD, Thomson MA, Davis DA, Haynes RB (1995). No magic bullets: A systematic review of 102 trials of interventions to improve professional practice. CMAJ.

[B28] Abelson J, Forest PG, Casebeer A, Mackean G, Effective Public Consultation Project Team (2004). Will it make a difference if I show up and share? A citizens' perspective on improving public involvement processes for health system decision-making. Journal of Health Services & Research Policy.

[B29] Golden-Biddle K, Reay T, Petz S, Witt C, Casebeer A, Pablo A, Hinings CR (2003). Toward a communicative perspective of collaborating in research: the case of the researcher-decision-maker partnership. Journal of Health Services & Research Policy.

[B30] Ross S, Lavis J, Rodriguez C, Woodside J, Denis JL (2003). Partnership experiences: Involving decision-makers in the research process. Journal of Health Services & Research Policy.

[B31] Walshe K, Rundall TG (2001). Evidence-based management: From theory to practice in health care. Milbank Quarterly.

[B32] Lomas J (2000). Using "Linkage and Exchange" to move research into policy at a Canadian Foundation. Health Affairs.

[B33] Lomas J, Sibbald W, Bion J (2000). Health services research: A domain where disciplines and decision makers meet. Evaluating Critical Care: Using Health Services Research to Improve Quality.

[B34] Lavis J, Robertson D, Woodside JM, McLeod CB, Abelson J, the Knowledge Translation Study Group (2003). How can research organizations more effectively transfer research knowledge to decision makers?. The Milbank Quarterly.

[B35] Delanty G (2001). Challenging knowledge: The university in the knowledge society.

[B36] Florida R, Cohen WM, Branscomb LM, Kodama F, Florida R (1999). Engine or infrastructure? The university role in economic development. Industrializing knowledge: University-Industry linkages in Japan and the United States.

[B37] Van Looy B, Callaert J, Debackere K (2006). Publication and patent behavior of academic researchers: Conflicting, reinforcing or merely co-existing?. Research Policy.

[B38] Fleuren M, Wiefferink K, Paulussen T (2004). Determinants of innovation within health care organizations. International Journal for Quality in Health Care.

[B39] Greenhalgh R, Robert G, MacFarlane F, Bate P, Kyriakidou O (2004). Diffusion of innovations in service organizations: Systematic review and recommendations. The Milbank Quarterly.

[B40] Thompson GN, Estabrooks CA, Degner LF (2006). Clarifying the concepts in knowledge transfer: A literature review. Journal of Advanced Nursing.

[B41] Jacobson N, Butterill D, Goering P (2004). Organizational factors that influence university-based researchers' engagement in knowledge transfer activities. Science Communication.

[B42] National Forum on Health (1997). Canada health action building on the legacy.

[B43] Canadian Institutes of Health Research Knowledge translation strategy: Niche and focus 2005–2009. http://www.irsc.gc.ca/e/24471.html.

[B44] Canadian Health Services Research Foundation Knowledge transfer and exchange. http://www.chsrf.ca/knowledge_transfer/index_e.php.

[B45] Canadian Health Research Institutes Knowledge translation (KT) & commercialization. http://www.cihr-irsc.gc.ca/e/29529.html.

[B46] Canadian Health Research Institutes President's message to the research community – January 2007. http://www.cihr-irsc.gc.ca/e/33080.html.

[B47] Graham I Knowledge translation: Making health research work for Canadians. http://www.cihr-irsc.gc.ca/e/33747.html.

[B48] Grimshaw JM, Thomas RE, MacLennan G, Fraser C, Ramsay CR, Vale L, Whitty P, Eccles MP, Matowe L, Shirran L, Wensing M, Dijkstra R, Donaldson C (2004). Effectiveness and efficiency of guideline dissemination and implementation strategies. Health Technol Assess.

[B49] Newton MS, Estabrooks CA, Norton P, Birdsell JM, Adewale AJ, Thornley R (2007). Health researchers in Alberta: An exploratory comparison of defining characteristics and knowledge translation activities. Implement Sci.

[B50] Merton RK (1973). The sociology of science: Theoretical and empirical investigations.

[B51] Estabrooks CA, Norton P, Birdsell JM, Newton MS, Adewale AJ, Thornley R Career costs in a new health research paradigm? Mode I and Mode II activity among health researchers in Alberta. Research Policy; under review.

[B52] Newton MS (2006). Knowledge translation in the mental health field: What does it involve and why does it matter?.

[B53] Newton MS, Klassen TP, Osmond MH, Thomson D (2007). Knowledge translation in child health: Concepts and challenges. invited panel, Knowledge for Child Health, Ottawa, ON: 5th Canadian Cochrane Symposium.

[B54] SEARCH Canada. http://www.searchca.net/users/folder.asp.

[B55] EXTRA. http://www.chsrf.ca/extra/index_e.php.

[B56] Di Blasi Z, Harkness E, Ernst E, Georgiou A, Kleijnen J (2001). Influence of context effects on health outcomes: A systematic review. Lancet.

[B57] Dopson S, Fitzgerald L (2005). Knowledge to action? Evidence-based health care in context.

[B58] Scott-Findlay S, Golden-Biddle K (2005). Understanding how organizational culture shapes research use. J Nurs Adm.

[B59] Thomson O'Brien MA, Oxman AD, Haynes RB, Davis DA, Freemantle N, Harvey EL (2000). Local opinion leaders: effects on professional practice and health care outcomes. Cochrane Database Syst Rev.

